# Fewer culturable *Lactobacillaceae* species identified in faecal samples of pigs performing manipulative behaviour

**DOI:** 10.1038/s41598-023-50791-0

**Published:** 2024-01-02

**Authors:** Emilia König, Paulina Heponiemi, Sanni Kivinen, Jaakko Räkköläinen, Shea Beasley, Tuomas Borman, Maria Carmen Collado, Vilja Hukkinen, Jouni Junnila, Leo Lahti, Marianna Norring, Virpi Piirainen, Seppo Salminen, Mari Heinonen, Anna Valros

**Affiliations:** 1 Research Centre for Animal Welfare, Department of Production Animal Medicine, University of Helsinki, 00790 Helsinki, Finland; 2 Functional Foods Forum, University of Turku, 20520 Turku, Finland; 3 Vetcare Ltd., 04600 Mäntsälä, Finland; 4 Department of Computing, University of Turku, 20500 Turku, Finland; 5 Institute of Agrochemistry and Food Technology-National Research Council (IATA-CSIC), 46980 Paterna, Valencia Spain; 6 EstiMates Ltd., 20520 Turku, Finland; 7 Present Address: Sheaps Oy, 03250 Ojakkala, Finland

**Keywords:** Microbiology, Animal behaviour

## Abstract

Manipulative behaviour that consists of touching or close contact with ears or tails of pen mates is common in pigs and can become damaging. Manipulative behaviour was analysed from video recordings of 45-day-old pigs, and 15 manipulator-control pairs (n = 30) were formed. Controls neither received nor performed manipulative behaviour. Rectal faecal samples of manipulators and controls were compared. 16S PCR was used to identify *Lactobacillaceae* species and 16S amplicon sequencing to determine faecal microbiota composition. Seven culturable *Lactobacillacea*e species were identified in control pigs and four in manipulator pigs. Manipulators (p = 0.02) and females (p = 0.005) expressed higher *Lactobacillus amylovorus*, and a significant interaction was seen (sex * status: p = 0.005) with this sex difference being more marked in controls. Females (p = 0.08) and manipulator pigs (p = 0.07) tended to express higher total *Lactobacillaceae*. A tendency for an interaction was seen in *Limosilactobacillus reuteri* (sex * status: p = 0.09). Results suggest a link between observed low diversity in *Lactobacillaceae* and the development of manipulative behaviour.

## Introduction

Different forms of damaging biting behaviour are common in pigs^1^, and can for instance be seen in psychologically stressed animals^2^. Biting behaviour is typically directed to tails and ears^1,3,4^. Four types of tail biting have been suggested: (1) obsessive, (2) sudden and forceful, (3) two-stage^5^, and (4) epidemic tail biting^6^. Tail biting decreases the welfare of animals^7^ and affects the farmer’s income^8^. The precise mechanism behind tail biting remains poorly understood, but research suggests that it is a multifactorial problem^8^. Chronic stress, salt deficiency, nutritional deprivation^3^, poor access to feed^7^, diet formulation, health problems, environmental discomfort, high stocking density, and inadequate rooting material^8^ are among the known risk factors for tail biting. Pig-directed manipulative behaviour can be defined as manipulation of tails/ears without always resulting in visible wounds^9^. For example, tail-in-mouth behaviour is linked to damaging behaviour and often precedes tail biting^10^. Manipulative and damaging behaviour are thus probably behaviours caused by similar motivational factors but expressed in different severity ranging from mild occasional manipulation to outbreaks with severely damaged ears and tails^8^. Current research suggests a relationship between negative behavioural expression and the gut microbiota. Van der Eijk and colleagues^11^, for instance, showed feather-pecking hens to have a lower abundance of *Lactobacillus* than their non-feather-pecking peers. Brunberg and colleagues^12^ suggested a link between tail biting in pigs and the gut microbiota composition, which was supported by recent studies that found structural^13^ and compositional^13,14^ differences in microbiota between tail biters and control groups.

*Lactobacillaceae* play an important role in modifying the host epigenome and in producing beneficial metabolites such as short-chain fatty acids and vitamins^15^. Microbial development is rapid in early life^16^, requiring adequate environmental exposure for modulating the host immunity^17^. Vertical transmission of microbes is known to begin in some species, e.g. cattle, equine, and human, during pregnancy^18–21^, and to continue during lactation^22–24^. Colostrum is a key contributor to faecal *Lactobacillaceae* composition, influencing it long-term^25,26^. Besides diet, medication has a long-term impact on alpha diversity (Shannon diversity and observed species^22^ and Faith’s phylogenetic diversity^27^) and can lead to its depletion^28^. A decreased diversity may increase susceptibility to infections, e.g. due to overgrowth of opportunistic microbes in the gut^28^.

As microbial metabolites play a central role in the interaction between microbes and their hosts^29^, the gut microbiota influences the brain via the microbiota–gut–brain axis^30^ by the vagus nerve, circulatory system, and immune system^30,31^. Interestingly, microbial metabolites have been shown to influence depression in humans through multiple pathways^32^. The microbiota produces^30,33,34^ and regulates^31^ neurotransmitters such as serotonin^35^. Varying serotonin concentrations have been associated with anxiety and aggressiveness in different species^36,37^. In pigs, low concentrations have been associated with pessimistic-like behaviour^38^, and lower whole blood and platelet serotonin levels have been associated with tail biters compared to neutral, victim or biter/victim pigs^39^. Further, Valros and colleagues^40^ found evidence of a tendency for increased serotonin metabolism in tail-biting pigs, while other studies failed to support this^41^. Extensive research is required to help us to better understand the interaction between behaviour and intestinal microbiota and to find ways to alleviate tail biting in pigs.

Our aim was to identify pigs that perform tail and/or ear manipulation (manipulator pigs) and to compare their faecal microbiota with that of control pigs not manifesting such behaviour. We expected differences in the faecal microbiota between these two behavioural phenotypes. Specifically, we hypothesized finding less culturable *Lactobacillaceae* species in manipulator pigs. Furthermore, we hypothesized observing decreased total alpha diversity (based on Shannon and Chao1 indices and observed species richness) of microbiota in manipulator pigs compared with control pigs.

## Results

### Observed behaviour of pigs

Manipulator pigs (n = 15) performed on average two (range 1–3) different manipulative behaviours during the total of 66 min observations per pig (Supplementary Table S1), mainly directed towards ears. While all manipulator pigs manipulated ears, only two of them bit the tails of their pen mates (Supplementary Table S1). Five manipulator pigs received on average one (range 1–2) type of manipulative behaviour during the 66-min observation time. All performed and received manipulative behaviours of the study pigs are listed in Supplementary Table S1. None of the control pigs performed or received manipulative behaviour during the observation time.

### Faecal *Lactobacillaceae* composition

The following seven culturable *Lactobacillaceae* species were identified in control pigs (in descending order of prevalence): *Lactobacillus amylovorus*, *Limosilactobacillus reuteri*, *Lactobacillus johnsonii*, *Limosilactobacillus mucosae, Lactobacillus delbrueckii, Limosilactobacillus pontis,* and *Ligilactobacillus salivarus.* Four species were identified in both manipulator and control pigs (in descending order of prevalence): *L. amylovorus*, *L. reuteri*, *L. johnsonii*, and *L. mucosae*. Control pigs tended (t_28_ = 1.7, p = 0.099) to have a higher average number of identified *Lactobacillaceae* species (1.8 species, SD 0.78) than manipulators (1.3 species, SD 0.72).

**Figure 1:**
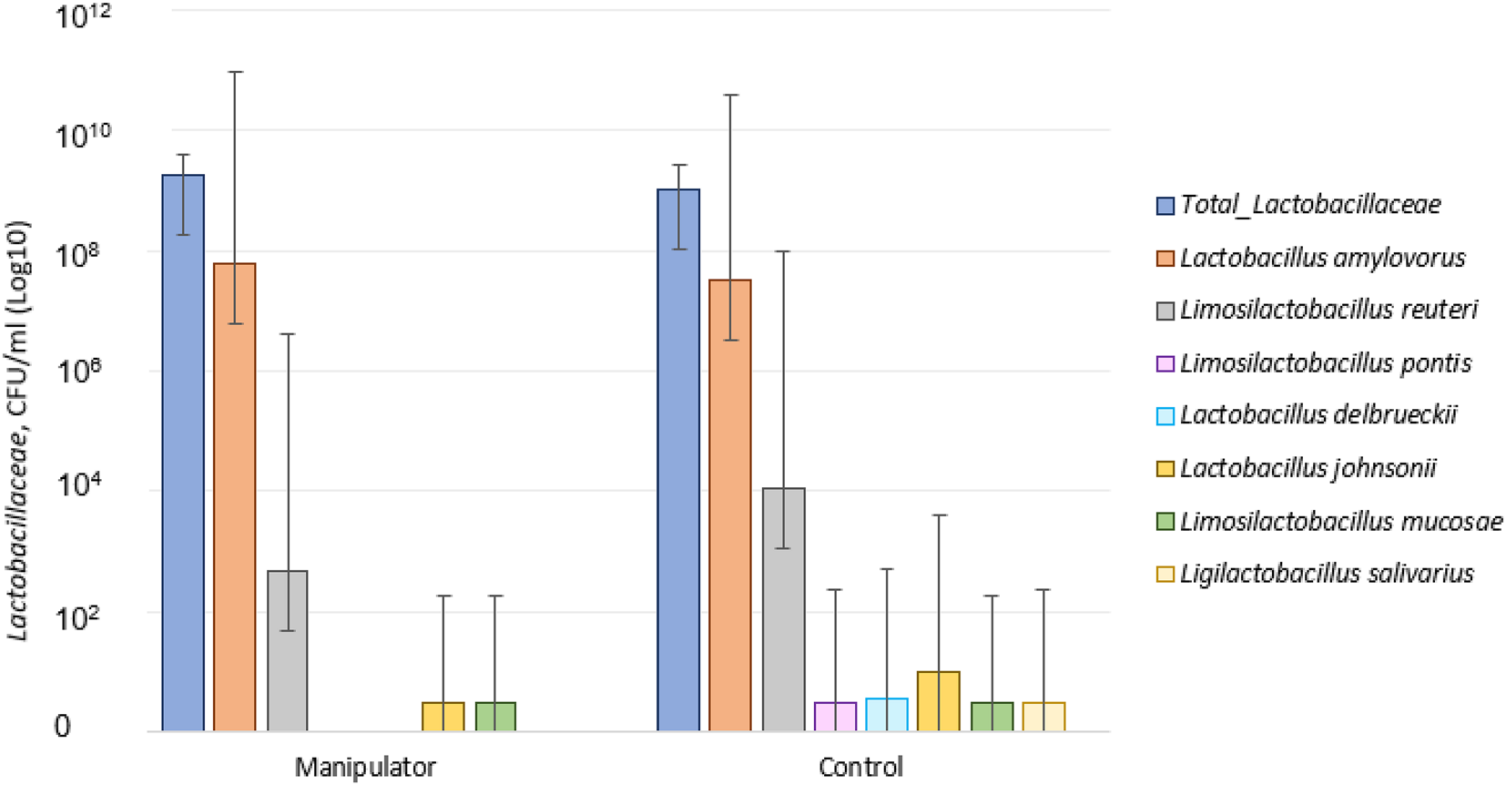
Mean number (and standard deviation of the values) of culturable *Lactobacillaceae* species composition in manipulator (n = 15) and control (n = 15) pigs shown on a log scale (p > 0.1).

In total, 50% of isolated *Lactobacillaceae* remained unidentified in manipulator pigs and 54% in control pigs. Overall, manipulator pigs had a higher number of total *Lactobacillaceae* species (mean 2.3 × 10^9^ colony-forming units (CFU)/ml; range 2 × 10^8^–5.6 × 10^9^) than control pigs (mean 1.2 × 10^9^ CFU/ml; range 1.4 × 10^8^–3.8 × 10^9^) (Fig. 1). *L. amylovorus* was identified in 87% and *L. reuteri* in 43% of the samples regardless of the behaviour of the pig. Other *Lactobacillaceae* species were identified in 3–10% of samples, with the control group expressing higher numbers of these *Lactobacillaceae* (Fig. 1). Based on the Mann–Whitney U-test, no significant differences (p > 0.1) were found in abundance of *L. johnsonii, L. mucosae, L. delbrueckii, L. pontis,* or *L. salivarius* between manipulator and control pigs (Fig. 1).

According to the linear mixed models, both female (F_1,26_ = 3.3, p = 0.08, female 1.98 × 10^6^ CFU/ml (SE 5.01 × 10^5^) vs. barrow 1.22 × 10^6^ CFU/ml (SE 4.49 × 10^5^)) and manipulator pigs (F_1,26_ = 3.6, p = 0.07, manipulator 1.99 × 10^6^ CFU/ml (SE 4.68 × 10^5^) vs. control 1.21 × 10^6^ CFU/ml (SE 4.8 × 10^5^)) tended to have higher colony counts of total *Lactobacillaceae* species.

**Figure 2:**
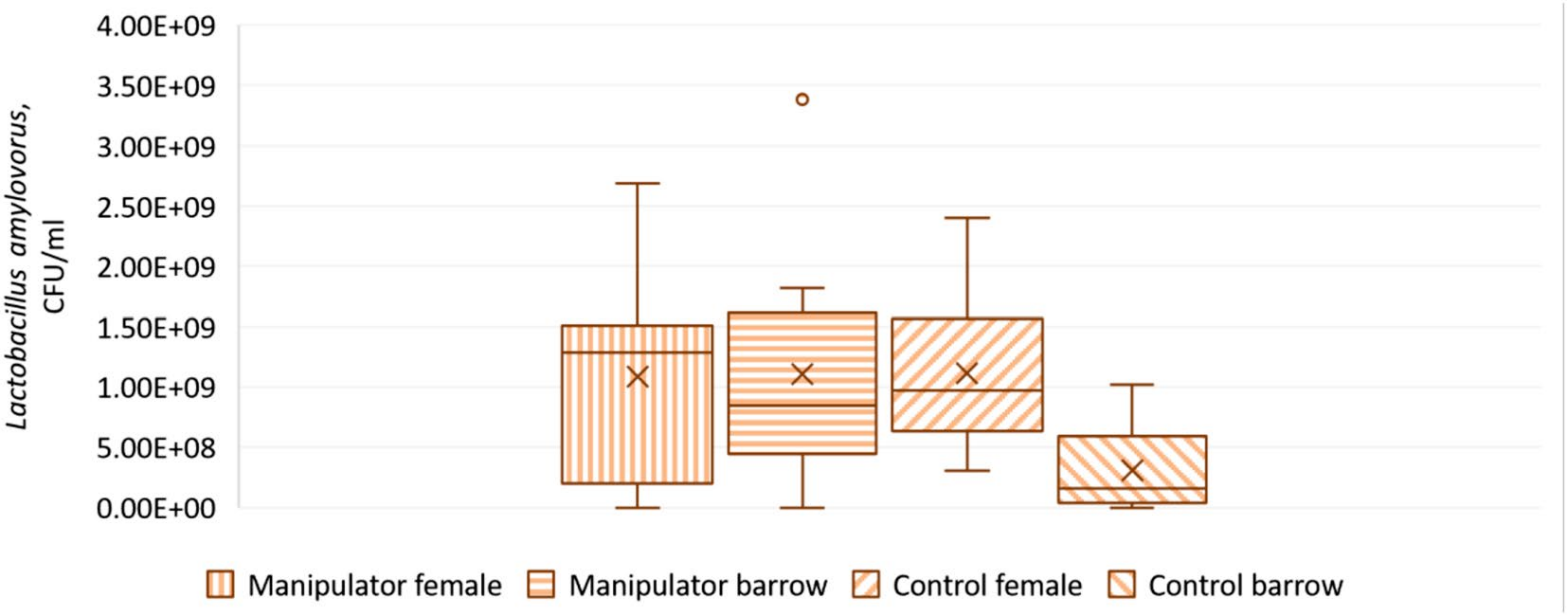
Amount of faecal *Lactobacillus amylovorus* in manipulator and control pigs. Back-transformed EM means of *L. amylovorus* (with 95% confidence interval) in manipulator (n = 15) and control pigs (n = 15) of both sexes (Linear mixed model, interaction sex * status: F_1,18_ = 9.9, p = 0.005). Middle line shows median, x shows the mean, dots show outliers, and whiskers show minimum and maximum values.

**Figure 3:**
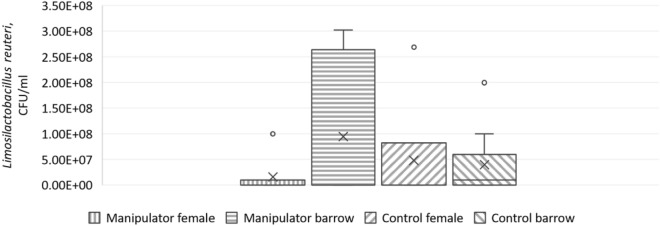
Amount of faecal *Limosilactobacillus reuteri* in manipulator and control pigs. Back-transformed EM means of *L. reuteri* (with 95% confidence interval) in manipulator (n = 15) and control pigs (n = 15) of both sexes (Linear mixed model, interaction sex * status: F_1,9_ = 3.6, p = 0.09). Middle line shows median, x shows the mean, dots show outliers, and whiskers show minimum and maximum values.

Female (F_1,18_ = 10.0, p = 0.005) and manipulator pigs (F_1,17_ = 6.5, p = 0.02) expressed higher colony counts of *L. amylovorus* than barrows and control pigs. In addition, a significant interaction sex * status (F_1,18_ = 9.9, p = 0.005) was seen (Fig. 2). The linear mixed model for *L. reuteri* showed a tendency for an interaction sex * status (F_1,9_ = 3.6, p = 0.09) (Fig. 3).

### Microbiota analysis

**Figure 4:**
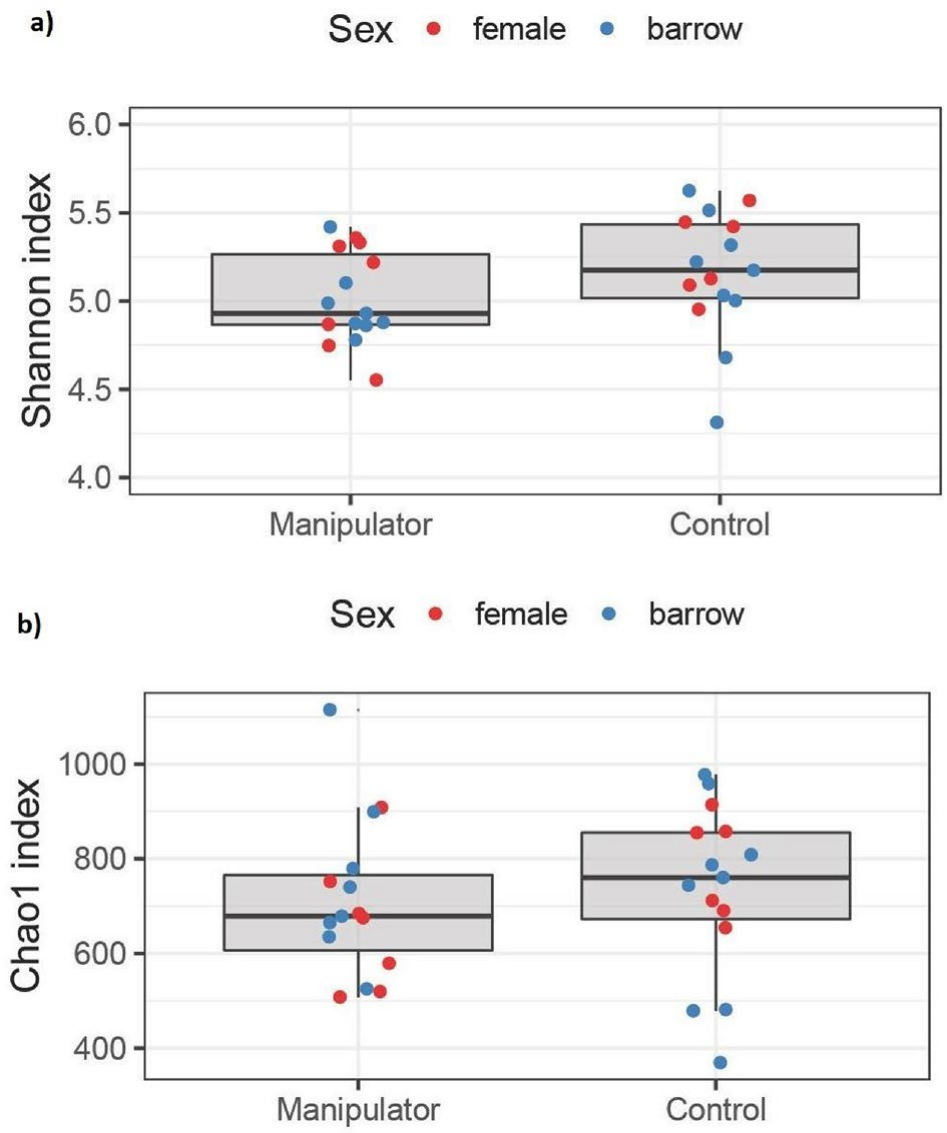
Total microbiota alpha diversity index. Microbiota (**a**) diversity (Shannon index: t_26_ = 1.22, p = 0.23) and (**b**) richness (Chao1 index: t_26_ = 1.23, p = 0.23) of manipulator (n = 15) and control (n = 15) pigs.

No statistical differences in alpha diversity were observed between manipulator and control pigs (Shannon index: t_26_ = 1.22, p = 0.23; and Chao1 index: t_26_ = 1.23, p = 0.23, Fig. 4).

**Figure 5:**
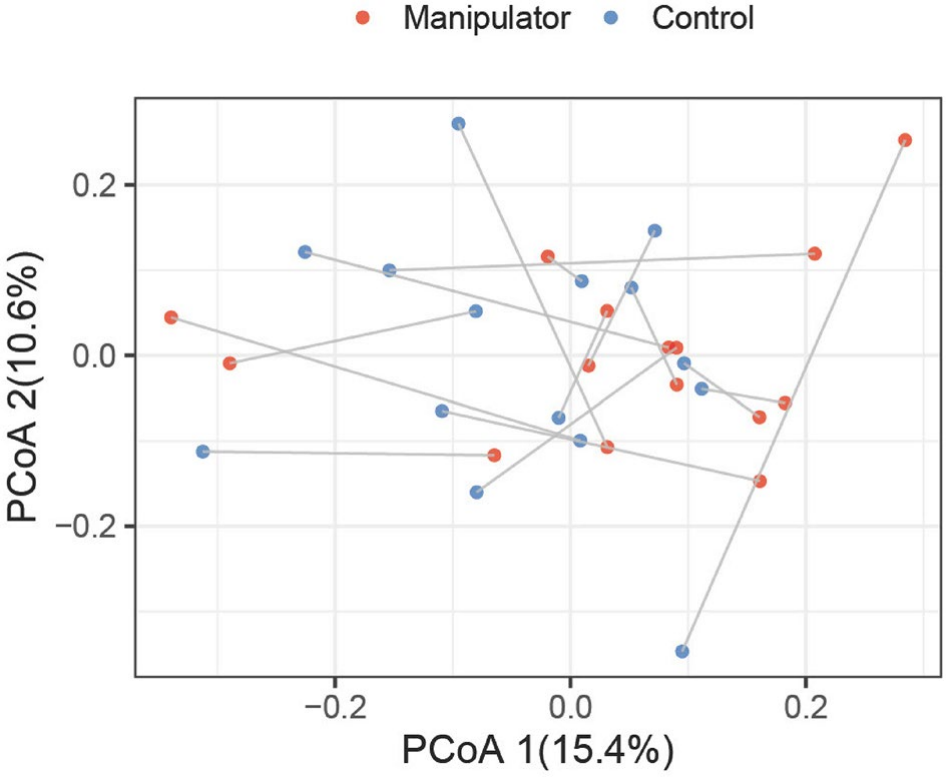
Sample similarity based on total community composition. Principal coordinates analysis (PCoA) with Bray–Curtis dissimilarity illustrates sample similarity based on amplicon sequence variant (ASV)-level taxonomic profiles (n = 30, PERMANOVA p = 0.26). The lines highlight matched control-manipulator pairs.

No significant differences were found in community composition, i.e. in beta diversity, between control and manipulator pigs (p = 0.26; PERMANOVA). The visualization of sample similarity with Principal Coordinates Analysis (PCoA; Bray–Curtis dissimilarity) further supported this result (Fig. 5). The control pigs show more variation along the second principal coordinate axis, indicating a more variable gut microbiota.

Amplicon sequence variant (ASV) was used to distinguish between sequences and to associate sequences with microbes. Of the 20 most important ASVs (detailed list in Supplementary Table S2) with respect to the differences between manipulator and control pigs, most were associated with the families *Lachnospiraceae* and *Ruminococcaceae* among the manipulator pigs and with the families *Bifidobacteriaceae*, *Christensenellaceae*, and *Lachnospiraceae* among the control pigs.

## Discussion

In this study, pigs at the age of 45 days with manipulative behaviour expressed a higher total abundance of faecal *Lactobacillaceae* species but a numerically lower alpha diversity. In a study by Rabhi and colleagues^13^, 13- to 17-week-old pigs of the non-biter/non-bitten negative control group harboured a significantly higher abundance of *Lactobacillus* than control pigs, whereas Verbeek and colleagues^14^ did not observe any difference in *Lactobacillus* between different tail biting phenotypes in their study with pigs aged over nine weeks. As the abundance of *Lactobacillaceae* tends to build in pigs through late pregnancy towards weaning^42^, disturbance during this development phase may result in pathology and disease in later life^42,43^. Shaping of the gastrointestinal microbiome occurs early in life^18,20,44,45^ and is mainly affected by environmental exposures and diet^46,47^.

Microorganisms belonging to the families *Lachnospiraceae* and *Ruminococcaceae* dominated numerically the faecal microbiota of manipulator pigs. *Ruminococcaceae* increase in pigs after weaning^48^ and are linked to plant carbohydrate degradation^49^. The faecal microbiota of control pigs was characterized by a higher abundance of *Bifidobacteriaceae*, *Christensenellaceae*, and *Lachnospiraceae*. These families have been associated with the preweaning period^18,48,49^. *Bifidobacteria* are associated with health benefits^50^, and they are commonly used as probiotics^51^. *Christensenellaceae* have recently moved into the focus of research, as *Christensenella minutia* has been suggested as a potential probiotic for human health and obesity^52^. In pigs, *Christensenellaceae* are linked to fermentation of sugars^49^.

A narrow range of *Lactobacillaceae* species has been documented in commercial pigs^53^, whereas wild boars showed higher alpha diversity than commercial pigs^54^. This tends to hold true in general; wild Kenyan warthogs scored higher in microbial richness than warthogs kept in captivity (zoo) and higher in alpha diversity than commercial pigs^55^. This reflects a similar phenomenon in other wild animal species compared with domesticated or semi-domesticated animals^54,56,57^, indicating higher microbial diversity in non-domesticated animals. In our study, the alpha diversity indices were numerically higher in control pigs, but the difference was not significant. However, our results suggest that a larger sample group could resolve this question. Especially in the female group, for which a subgroup analysis was done (results not presented, p = 0.12), the difference in alpha diversity could potentially become significant by increasing the number of samples.

Sex of the pig proved to be an important factor regarding *Lactobacillaceae*. A significant interaction with behaviour type was seen for *L. amylovorus* as well as a tendency for *L. reuteri*. Sex-related differences regarding tail biting have been shown previously; barrows seem to have a higher frequency of damaged tails than females, but it has not yet been clearly shown that one sex is more prone to perform tail biting^8^. The influence of sex on microbial composition has been overlooked in previous years, although the topic is gaining in awareness^58^. Interestingly, Verbeek and others^14^ found a significant main effect of sex in faecal short-chain fatty acid compositions, which is most probably due to an underlying difference in gastrointestinal microbiota. The effect of sex on microbial diversity was not the focus of this study, but the reasons for differences in faecal *Lactobacillaceae* composition between females and barrows warrant further research.

A species-specific microbiota composition contributes to animal health and development, with acquisition of a specific selection of microbes resulting in the highest benefit for the host^59^. Gut microbiota is known to regulate host gene expression^60^, and therefore, an undisturbed diverse microbiota contributes to richness of metabolites in the host providing several health benefits^61^. The gut microbiota functions as an endocrine organ^62^, and it plays a crucial role in the synthesis and regulation of neurotransmitters, e.g. serotonin^63^. As the evolving microbiota may show low abundance and observed diversity of *Lactobacillaceae,* a reduced or delayed development of the gut microbiota may be linked to psychological stress^64–66^, potentially causing pig-directed manipulative behaviour. Also, exposure to social stressors has been shown to reduce alpha diversity^7^, while the gut microbiota can influence the host’s susceptibility to stress^67^. Tail biting and manipulative behaviour are both suggested to be caused by stress^8^ and further to create stress in pigs receiving these behaviours^3,68^. This study, however, does not allow for conclusions on causal relationships or their directions.

Mild manipulation, i.e. no visible harm to tails, in young pigs can turn into damaging tail biting in older pigs^10,69^. For example, sickness has been shown to lead to manipulative behaviour in pigs^69^. Nordgreen and others^2^ have suggested a link between compromised health and damaging behaviour, which may be mediated through the microbiota-gut-brain axis. A high abundance of *Lactobacillaceae* does not necessarily equal better health, but high diversity and richness have been shown to be associated with better health^23^ and are suggested to offer the host a more flexible response to environmental changes^70^. Le Chatelier and colleagues^71^ reported a low number of gut microbial genes (i.e. a low gene count) to be associated with an unhealthy state, as characterized by higher adiposity, insulin resistance, dyslipidaemia, and low-grade inflammation. Also, Dao and colleagues^72^ and Cotillard and colleagues^73^ have confirmed the positive correlations between a high basal gene count, healthier metabolic status, and better outcomes after dietary restriction, supporting the importance of the evaluation of gene richness.

Regarding the limitations of this study, the small sample size remains the biggest limitation. Even though a large number of individually ear-tagged pigs were included to begin with, only 15 manipulators and 15 controls could be reasonably matched. We recognize, that by including as few as 30 pigs into the study, the sample size is quite small yet comparable to similar studies^13,14^. Further, we compared only two groups in this study. It was not possible for us to form a separate victim-group due to manipulator pigs in many cases being also victims. Recognizing control pigs was also difficult. The behaviour of manipulator and control pigs may not have been sufficiently different from each other to see significant differences in the microbiota. On the other hand, trends observed in this setting could point towards even greater microbial differences during an actual tail-biting episode, which would make it possible to identify more extreme behavioural phenotypes. Secondly, we included only one farm in the study to focus on microbial differences in individuals. This farm was chosen based on frequent tail biting outbreaks in the past. Despite of the history of the farm, no severe lesions were seen on the pigs and mainly ear manipulation was observed and analysed. Thirdly, we acknowledge, that choosing manipulators and controls from the same pens increased the risk of microbes transferring between pen mates since the manipulator-control pairs ate the same feed, were kept under the same conditions, and most of the pairs were within transmission distance. Both Rabhi and colleagues^13^ and Verbeek and colleagues^14^ chose biters and controls from different pens. By choosing both manipulator and control primarily form the same pen, our study setup enabled separating the effect of pen and phenotype, which differs from previous studies. Finally, the laboratory methods used may have been a further limiting factor since many of the *Lactobacillaceae* species could not be identified, instead being included in the total *Lactobacillaceae* count. The confidence of identification of microbes relies on alignment to a limited set of sequences from culture collections^74^. Additionally, amplification of the V3-V4 variable region of the 16S rRNA gene does not provide adequate taxonomic resolution to reliably identify bacteria at species or strain level^75^. Despite of these limiting factors, we were able to pinpoint some significant differences.

In conclusion, these results indicate an association between the faecal microbiota and pig-directed manipulative behaviour while not allowing for conclusions on causality. The difference in microbial composition shows promising trends, warranting further studies. These results suggest that low observed diversity in *Lactobacillaceae* and the development of manipulative behaviour in pigs may be linked. The link between total faecal microbiota, especially beneficial microbes, and their metabolism, and the development of manipulative behaviour may be better disclosed with a larger sample size and sampling during an ongoing tail biting episode, allowing for the identification of more extreme behavioural phenotypes.

## Methods

### Ethical statement

The institutional review board of the Regional State Administrative Agency for Southern Finland approved the experiment and the experimental protocol (ESAVI/16950/2018). All experiments were performed in accordance with relevant guidelines and regulations. Reporting in the manuscript follows the ARRIVE guidelines recommendations.

### Animals and housing

The study was performed on a commercial farm in South-West Finland in November 2019. The farm was chosen due to a history of frequent tail biting outbreaks. Study pigs were chosen from all piglets (Norwegian Landrace x Norwegian Yorkshire) born on the farm within seven days. Piglets (n = 478) were individually ear-tagged at birth and were visually assigned to three size categories: small, medium, or large. Animal caretakers castrated all male piglets surgically before the age of seven days and relieved their castration pain with intramuscularly injected meloxicam prior to and one day after the castration. No tail docking or teeth clipping was done.

Piglets were housed with their dams in farrowing pens with farrowing crates until weaning at the mean age of 25 (range 23–27) days. Thereafter, piglets were moved to the weaning facilities, with each pen holding 15–22 piglets. A mean of 21 study pigs (range 1–22) were housed in one weaning pen together with non-study pigs in two climate-pens with total space of 10.6 m^2^. Pens had an elevatable roof over the lying area. Approximately 22% of the pen area was a partly slatted concrete floor. The pigs were provided with one iron chain per pen. Caretakers distributed sawdust, straw, or peat weekly. At least small amounts of these materials were always visible in the pen. Two pens shared a five-m-long feeding trough, and the pigs were liquid fed according to Finnish standards. Pigs had ad libitum access to water from three nipples/pen next to the feeding trough.

### Faecal sampling

Faecal samples were collected from study pigs one day before the start of video recording. One researcher held the pigs while another collected a faecal rectal sample with a clean disposable glove and placed it into a faecal sampling tube. Tubes were stored in cooling boxes, moved to – 20 °C within an hour, and transported to – 80 °C at the end of the collection day.

### Video recording and behavioural analysis

At the start of the observation period, the mean age of the pigs was 45 (range 43–47) days. Six cameras (IP Camera, IPC-HFW1230S-0280B-BLACK, Zhenjiang Dahua Vision Technology Co., Ltd., Hangzhou, P.R. China) were fixed on the ceiling so that one camera filmed two pens. Recordings were managed from a connected computer with surveillance software (Blue Iris Security, Video Management Software, https://blueirissoftware.com/). Pigs were spray-marked on the back for individual recognition in pens/on video recordings. Markings were reinforced every other day at noon.

The pens were video-recorded for eight consecutive days. Footage from days one, three, and seven was selected for analysis because pigs were marked on these days and to achieve as long a time span as possible. The times of high behavioural activity around feeding were selected for observations. On each of the three days, late afternoon feeding (at approximately 5:30 p.m.) and evening feeding (at approximately 10 p.m.) were observed. These feedings were after the end of the working day of the caretakers, ensuring that the pigs were not disturbed or startled. Observations started one minute before the pigs received feed and lasted for eleven minutes. This adds up to 66 min of observation for each pig. One person analysed all the chosen recordings with a one-zero protocol. After observing the behaviour of each pig for one minute at a time (within the timeframe of eleven minutes/feeding), it was recorded whether the pig performed or received any of the behaviours listed in the ethogram presented in Table 1.

**Table 1:** Ethogram of behaviour categorized as manipulative behaviour.

Manipulative behaviour	Definition
Tail manipulation	Snout in touching distance to tail, or nosing around tail area, or chewing on tail. Recipient does not react
Tail biting	Snout in touching distance to tail, or nosing around tail area, or chewing on tail. Recipient reacts physically by walking away, shaking the head, or retaliating
Ear manipulation	Snout in touching distance to ear, or nosing around ear area, or chewing on ear. Recipient does not react
Ear biting	Snout in touching distance to ear, or nosing around ear area, or chewing on ear. Recipient reacts physically by walking away, shaking the head, or retaliating

Touching distance = possibility to hold tail or ear in mouth.

### Selection of manipulator and control pigs

The ear-tagged pigs were divided into two rooms within the weaning facilities. A total of 210 pigs were selected from one room for video recording. The selected room contained twelve pens, of which ten pens, containing more than ten study pigs, were chosen. Pigs with one or more records of performing manipulative behaviour on at least two out of the three observation days were defined as manipulators (n = 20). Similarly, pigs with no records of performed or received manipulation were defined as control pigs (n = 21). These pigs were matched to 15 manipulator-control pig pairs as shown in Table 2. Pairs were standardized as far as possible based on sex, birth week size, and pen. At least two of these attributes had to match for the pairs to be included in the study. In the final dataset, we had 17 male and 13 female pigs. Out of these 30 pigs, two were categorized as small, 23 as medium, and five as large at birth.

**Table 2:** Manipulator-control pig pairs showing the outcome of the matching process.

	Pen	Birth size	Sex
Five manipulator-control pig pairs	Same	Same: one large pair, four medium pairs	Same: three male pairs, two female pairs
One manipulator-control pig pair	Same	Different: one large pig + medium pig pair	Same: both female
Five manipulator-control pig pairs	Same	Same: one large pair, four medium pairs	Different: male + female pig, five pairs
Four manipulator-control pig pairs	Different	Same: one small pair, three medium pairs	Same: three male pairs, one female pair

### Lactobacillaceae analysis

Determination of faecal *Lactobacillaceae* was done as previously described by König and colleagues^26^. Briefly, faecal samples were thawed on ice and a tenfold dilution series was plated on blood liver (BL) agar. All non-identical colonies were picked from the plate, moved into Gifu Anaerobic Medium Broth, and incubated. Under microscope, non-motile rod-shaped bacteria were streaked on BL agar plates, and their DNA was isolated. Different *Lactobacillaceae* were identified with a colony PCR, and *Lactobacillaceae* species were identified with a 16S PCR. The PCR product was purified, the DNA measured and sequenced, and *Lactobacillaceae* species were identified with the National Center for Biotechnology Information’s (NCBI) Basic Local Alignment Search Tool (BLAST) database with 96% accuracy.

### 16S rRNA amplicon sequencing

Faecal samples were weighed (0.100–0.125 g) into a two ml screw cap tube and microbiota analysis was performed with InviMag^®^ Stool DNA Kit (Invitek Molecular GmbH, Berlin, Germany) according to the manufacturer’s instructions. A FastPrep^®^-24 Sample Preparation System (M.P. Biomedicals, Irvine, California, USA), a Heraeus Pico 17 Centrifuge (Thermo Fisher Scientific Ltd., Osterode am Harz, Germany), and a KingFisher™ Purification System Type 700 (Thermo Fisher Scientific Ltd., Vantaa, Finland) were used for the extraction. DNA concentrations of eluates were measured with a NanoDrop 2000 UV–Vis Spectrophotometer (Thermo Fisher Scientific Ltd., Wilmington, Delaware, USA).

DNA concentration was measured using a Qubit^®^ 2.0 Fluorometer (Life Technology, Carlsbad, California, USA) to normalize concentrations to run the libraries. DNA libraries were performed with amplification of the V3-V4 variable region of the 16S rRNA gene with the primers selected from Klindworth and colleagues^76^, as described previously^77^. A multiplexing step was conducted with the NextEra XT Index Kit (FC-131-2001) (Illumina, San Diego, California, USA), and DNA quality of the library PCR product was measured with a Bioanalyzer DNA 1000 chip (Agilent Technologies, Santa Clara, California, USA) to verify the size (expected size on a Bioanalyzer trace is ~ 550 bp). The libraries were sequenced using a 2 × 300 bp paired-end run on MiSeq Illumina platform according to the manufacturer’s instructions.

The 16S rRNA amplicon sequences were pre-processed with the DADA2^78^ algorithm (R package dada2^79^), by customizing an established workflow^80^. The sequences were truncated based on manual investigation of the quality plots (forward 260; reverse 210) and trimmed from the left (base 19). Reads with more than two expected errors were discarded. Otherwise, default settings were used. Chimeric sequences were removed. The ASVs were inferred from the processed sequences. The taxonomic assignment of the ASVs was done against the Silva database (version 138.1)^81^ downloaded at the URL https://benjjneb.github.io/dada2/training.html (accessed 22 Feb 2022). The abundance tables, taxonomic mappings, and phenotype data were assembled into the TreeSummarizedExperiment data container^82^.

### Data handling and statistical analysis

#### Lactobacillaceae

For statistical analysis, only clearly identified *Lactobacillaceae* were considered. Multiple possible species were regarded as unidentified *Lactobacillaceae* sp. Statistical analyses were done in SPSS (IBM Corp. released 2020. IBM SPSS Statistics for Windows, version 27.0. Armonk, New York, USA). All variables were checked for normal distribution, and only the total number of *Lactobacillaceae* was found to be normally distributed. A paired samples t-test was performed to check for differences in number of identified *Lactobacillaceae* species between manipulator and control pigs. To compare the amount of *Lactobacillaceae* between manipulator and control pigs, linear mixed models (LMM) were conducted. This was, however, possible only for *L. amylovorus*, *L. reuteri,* and total *Lactobacillaceae* since the other identified *Lactobacillaceae* species were too scarcely represented in the samples. For the LMM, both *L. amylovorus* and *L. reuteri* were log10-transformed to achieve close to normal distribution. Initially, all three models included piglet birth size and pen as random factors and sex and status (manipulator or control) as fixed factors. The interaction sex * status was tested for all models but was significant and improved model quality based on AIC value only in models for *L*. *amylovorus* and *L. reuteri*. Model residuals were checked for normal distribution. Differences between manipulator and control pigs in the amount of *Lactobacillaceae* species present in samples were checked with Mann–Whitney U-test.

#### Microbial diversity

To assess possible differences in manipulator and control pig microbial diversity the following analyses were performed. Taxonomic profiling data were analysed with the multiassay framework^83^ using the R package vegan^84^. A total read count of 0.95 million reads was retrieved for the 30 samples, with an average of 31,819 reads per sample (range 14 917–97 967 reads). These were assigned into 11,056 ASVs (141 singletons) from 36 phyla, 70 classes, 119 orders, 164 families, 351 genera, and 99 species. Alpha diversity was estimated using the Shannon and Chao1 indices. Two-way analysis of variance (ANOVA) models were constructed to compare the status groups and sex, with the main effects of status and sex together with their interaction term. Community composition (beta diversity) was visualized with Principal Coordinates Analysis (PCoA) based on Bray–Curtis dissimilarities. Permutational analysis of variance (PERMANOVA) was used to compare the status groups regarding beta diversity (R function vegan::adonis).

### Supplementary Information

Supplementary Tables.

## Data Availability

The 16S rRNA sequences for this study have been deposited with links to BioProject accession number PRJNA937729 in the NCBI BioProject database: https://www.ncbi.nlm.nih.gov/bioproject/PRJNA937729. The datasets generated during and/or analysed during the current study are available in the Github repository Manipulative_behaviour: https://github.com/emiliakonig/Manipulative_behaviour.
