# Supplementary material for: Fewer culturable Lactobacillaceae species identified in faecal samples of pigs performing manipulative behaviour
Source: Sci Rep. 2024 Jan 2;14:132. doi: 10.1038/s41598-023-50791-0 (PMC10762183; doi:10.1038/s41598-023-50791-0)
Supplement: Supplementary file 1 — Supplementary Tables. [file 41598_2023_50791_MOESM1_ESM.pdf]

Fewer culturable *Lactobacillaceae* species identified in fecal samples of pigs performing manipulative behaviour

Emilia König[1,*], Paulina Heponiemi[2], Sanni Kivinen[2], Jaakko Räkköläinen[2], Shea Beasley[3], Tuomas Borman[4], Maria Carmen Collado[5], Vilja Hukkinen[1], Jouni Junnila[6], Leo Lahti[4], Marianna Norring[1], Virpi Piirainen[1], Seppo Salminen[2], Mari Heinonen[1], Anna Valros[1]

[1]Research Centre for Animal Welfare, Department of Production Animal Medicine, University of Helsinki, 00790 Helsinki, Finland

[2]Functional Foods Forum, University of Turku, 20520 Turku, Finland

[3]Vetcare Ltd., 04600 Mäntsälä, Finland, Present affiliation: Sheaps Oy, 03250 Ojakkala, Finland

[4]Department of Computing, University of Turku, 20500 Turku, Finland

[5]Institute of Agrochemistry and Food Technology-National Research Council (IATA-CSIC), 46980 Paterna, Valencia, Spain

[6]EstiMates Ltd., 20520 Turku, Finland

*Corresponding author: emilia.konig@helsinki.fi

Manipulator pig	Intensity of performed manipulative behaviours					Intensity of received manipulative behaviours				
	Tail man.	Ear man.	Tail biting	Ear biting	Number of different types of manipulative behaviours	Tail man.	Ear man.	Tail biting	Ear biting	Number of different types of manipulative behaviours
**1**	-	++	-	+	2	-	+	-	-	1
**2**	-	+	-	+	2	-	-	-	-	0
**3**	-	+	+	-	2	-	+	-	-	1
**4**	-	++	-	-	1	-	++	-	-	1
**5**	-	+	-	-	1	-	-	-	-	0
**6**	-	+	-	++	2	-	-	-	-	0
**7**	+	+++	-	+++	2	-	-	-	-	0
**8**	+	+	-	-	2	+	-	-	-	1
**9**	+	+	-	-	2	+	-	-	+	2
**10**	+	+	-	+	3	-	-	-	-	0
**11**	+	+	-	-	2	-	-	-	-	0
**12**	+	++	-	-	2	-	-	-	-	0
**13**	-	+	-	-	1	-	-	-	-	0
**14**	-	+	+	+	3	-	-	-	-	0
**15**	-	+	-	+	2	-	-	-	-	0

Supplementary Table S1. Number of different performed and received manipulative behaviours and their intensity for each manipulator pig during the 66-minute observation period of video recordings.

man. = manipulation

Tail/ear manipulation = snout in touching distance to tail/ear, recipient does not react to manipulation

Tail/ear biting = snout in touching distance to tail/ear, recipient reacts physically by walking away, shaking the head, or retaliating

Total intensity of manipulation during total of 66-minute observation period (range 0-6): - = 0 manipulations, + = 1-2 manipulations, ++ = 3-4 manipulations, +++ = 5-6 manipulations

ASV	Phylum	Class	Order	Family	Genus	Species
**ASV1**	Firmicutes	Clostridia	Clostridiales	*Clostridiaceae*	*Clostridium sensu stricto 1*	NA
**ASV8**	Firmicutes	Clostridia	Lachnospirales	*Lachnospiraceae*	*Agathobacter*	NA
**ASV2**	Firmicutes	Clostridia	Peptostrepto-coccales-Tissierellales	*Peptostrepto-coccaceae*	*Terrisporobacter*	NA
**ASV17**	Firmicutes	Clostridia	Lachnospirales	*Lachnospiraceae*	*Agathobacter*	NA
**ASV16**	Firmicutes	Clostridia	Oscillospirales	*Ruminococcaceae*	*Faecalibacterium*	*prausnitzii*
**ASV95**	Firmicutes	Clostridia	Lachnospirales	*Lachnospiraceae*	*Agathobacter*	NA
**ASV44**	Firmicutes	Clostridia	Oscillospirales	*Ruminococcaceae*	*Faecalibacterium*	*prausnitzii*
**ASV154**	Firmicutes	Clostridia	Lachnospirales	*Lachnospiraceae*	*Roseburia*	NA
**ASV32**	Firmicutes	Clostridia	Oscillospirales	*Ruminococcaceae*	*Subdoligranulum*	NA
**ASV52**	Firmicutes	Clostridia	Lachnospirales	*Lachnospiraceae*	*Agathobacter*	NA
**ASV51**	Firmicutes	Clostridia	Oscillospirales	*Oscillospiraceae*	*UCG-002*	NA
**ASV232**	Actino-bacteria	Actino-bacteria	Bifidobacteriales	*Bifidobacteriaceae*	*Bifidobacterium*	NA
**ASV29**	Firmicutes	Clostridia	Christensenellales	*Christensenellaceae*	*Christensenellaceae R-7 group*	NA
**ASV14**	Firmicutes	Clostridia	Lachnospirales	*Lachnospiraceae*	*Blautia*	NA
**ASV15**	Firmicutes	Clostridia	Oscillospirales	*Butyricicoccaceae*	*NA*	NA
**ASV13**	Firmicutes	Clostridia	Oscillospirales	*Eubacterium] coprostanoligenes group*	*NA*	NA
**ASV193**	Actino-bacteria	Actino-bacteria	Bifidobacteriales	*Bifidobacteriaceae*	*Bifidobacterium*	NA
**ASV322**	Actino-bacteria	Actino-bacteria	Bifidobacteriales	*Bifidobacteriaceae*	*Pseudoscardovia*	*radai*
**ASV10**	Firmicutes	Clostridia	Christensenellales	*Christensenellaceae*	*Christensenellaceae R-7 group*	NA
**ASV47**	Firmicutes	Clostridia	Lachnospirales	*Lachnospiraceae*	*Agathobacter*	NA

Supplementary Table S2. The Amplicon sequence variants (ASV) that explain differences between manipulator and control pigs. ASV1 to ASV52 are elevated in control pigs and ASV51 to ASV47 are elevated in manipulator pigs.
NA = not available
